# Hepatoprotective Effects of MHY3200 on High-Fat, Diet-Induced, Non-Alcoholic Fatty Liver Disease in Rats

**DOI:** 10.3390/molecules23082057

**Published:** 2018-08-16

**Authors:** Min Jo Kim, Chan Hum Park, Dae Hyun Kim, Min Hi Park, Kyung Chul Park, Min Kyung Hyun, A Kyoung Lee, Hyung Ryong Moon, Hae Young Chung

**Affiliations:** 1 Molecular Inflammation Research Center for Aging Intervention (MRCA), College of Pharmacy, Pusan National University, Busan 609-735, Korea; kiki10304@gmail.com (M.J.K.); bioimmune@hanmail.net (D.H.K.); bb36509@naver.com (K.C.P.); mminn94@naver.com (M.K.H.); lak000@naver.com (A.K.L.); 2 Department of Medicinal Crop Research, National Institute of Horticultural and Herbal Science Rural Development Administration, Eumseong 27709, Korea; ptman123@naver.com; 3 Department of Pharmaceutical Sciences, Irma Lerma Rangel College of Pharmacy, Texas A&M Health Science Center, College Station, TX 77843, USA; minhipark@tamhsc.edu

**Keywords:** 2-(4-(5-chlorobenzo[*d*]thiazol-2-yl)phenoxy)-2,2-difluoroacetic acid (MHY3200), PPARα agonist, lipid accumulation, ER stress, insulin signaling, inflammation

## Abstract

This study investigated the effects of 2-(4-(5-chlorobenzo[*d*]thiazol-2-yl)phenoxy)-2,2-difluoroacetic acid (MHY3200) on high-fat diet (HFD)-induced hepatic lipid accumulation and inflammation. The measurement of peroxisome proliferator-activated receptor (PPAR)α activity by using a luciferase assay indicated that MHY3200 was more potent than a known PPARα agonist, WY14643, in AC2F cells. Six-month-old male SD rats were fed chow or HFD for 1 month, and after, with or without added MHY3200 (1 or 2 mg/kg/day) for 4 weeks. The oral administration of MHY3200 caused a significant decrease in serum triglyceride (TG), glucose, alanine aminotransferase, and insulin, as well as a slight decrease in the level of free fatty acid and aspartate transaminase. No weight gain was detected when compared with HFD rats, and hepatic TG content was also attenuated by the administration of MHY3200. Furthermore, phosphorylation of the ER stress marker, inositol-requiring kinase 1 and its downstream gene, c-Jun N-terminal kinase, in addition to serine phosphorylation of insulin receptor substrate 1 were suppressed by MHY3200. Consistent with these results, MHY3200 administration reduced the levels of activation of protein-1, cyclooxygenase-2, and inducible nitric oxide synthase. Our results suggested that MHY3200 ameliorated HFD-induced hepatic lipid accumulation and inflammation, and improved insulin resistance through PPARα activation.

## 1. Introduction

Nonalcoholic fatty liver disease (NAFLD) is one of the types of fatty liver disease which is a major public health problem in industrialized countries; 20% of the U.S. population is thought to have the disease [1,2]. It encompasses a spectrum of hepatic damage associated with lipid deposition. It progresses to steatosis (simple fatty liver) and non-alcoholic steatohepatitis (fatty changes with inflammation and hepatocellular injury or fibrosis) [3]. NAFLD is considered to be the hepatic component of metabolic syndromes as its features are similar to those of metabolic disorders, such as obesity, type 2 diabetes, insulin resistance, and inflammation [4].

Insulin resistance is a common feature of obesity that is associated with metabolic syndrome, including type 2 diabetes, hypertension, cardiovascular disease, as well as NAFLD [5,6]. Although it is unclear as to how obesity causes insulin resistance, it is known that endoplasmic reticulum (ER) stress is related to the development of insulin resistance in obesity [7,8]. The ER stress is a central organelle and particularly susceptible to misfolded proteins [9]. Various studies have linked ER stress to abnormal insulin action and inflammation, and have suggested that ER stress activates the c-Jun N-terminal kinase (JNK) [10]. In addition, obesity-induced ER stress triggers the development of insulin resistance and diabetes through JNK phosphorylation via the inositol-requiring enzyme-1 (IRE-1), thereby inhibiting insulin receptor signaling [11]. Also, the activation of JNK triggers an accumulation of the c-Jun protein, inducing AP-1 transcriptional activity, resulting in inflammatory responses [12].

Peroxisome proliferator-activated receptors (PPARs) are well-known ligand-activated transcription factors of the nuclear receptor superfamily. The three subtypes of the PPAR family include peroxisome proliferator-activated receptor α (PPARα), PPARβ, and PPARγ [13]. PPARs regulate a variety of biological processes related to energy metabolism, including cell proliferation, differentiation, gluconeogenesis, insulin resistance, and lipid synthesis [14]. In the liver, activation of PPARα improves hepatic insulin resistance to elevated ER stress [15]. Additionally, up-regulation of PPARα stimulates the expression of genes involved in peroxisomal β-oxidation, thereby improving insulin sensitivity. In our previous studies, benzothiazole derivatives were synthesized and evaluated as potential PPARα agonists for the management of insulin resistance-related disorders, such as type 2 diabetes and aging. Docking simulations showed that the binding affinities of 2-[4-(5-chlorobenzothiazothiazol-2-yl)phenoxy]-2-methyl-propionic acid (MHY908) and 4-(benzo[*d*]thiazol-2-yl)benzene-1,3-diol (MHY553) were greater than those of fenofibrate, a selective PPARα activator [16,17]. MHY908 suppressed ER stress and consequently improved insulin resistance in type 2 diabetic *db/db* mice [16]. In addition, MHY553 alleviated hepatic steatosis through increased fatty acid oxidation and decreased inflammation in the aged rats [17].

Although the PPARα-agonistic activities of benzothiazole derivatives have been studied previously, PPARα-agonistic activities of MHY3200, a new synthetic benzothiazole derivative, have not been reported. Thus, the present study investigated the effects of MHY3200, a novel PPARα agonist, on lipid accumulation-independent ER stress and insulin resistance-induced inflammation in the livers of rats with high-fat diet-induced obesity.

## 2. Results

### 2.1. Effects of MHY3200 on PPARα Transcriptional Activity

**Figure 1:**
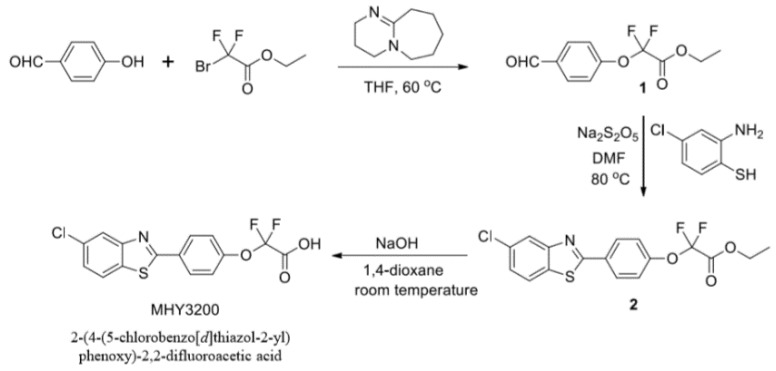
Synthesis of 2-(4-(5-chlorobenzo[*d*]thiazol-2-yl)phenoxy)-2,2-difluoroacetic acid (MHY3200).

**Figure 2:**
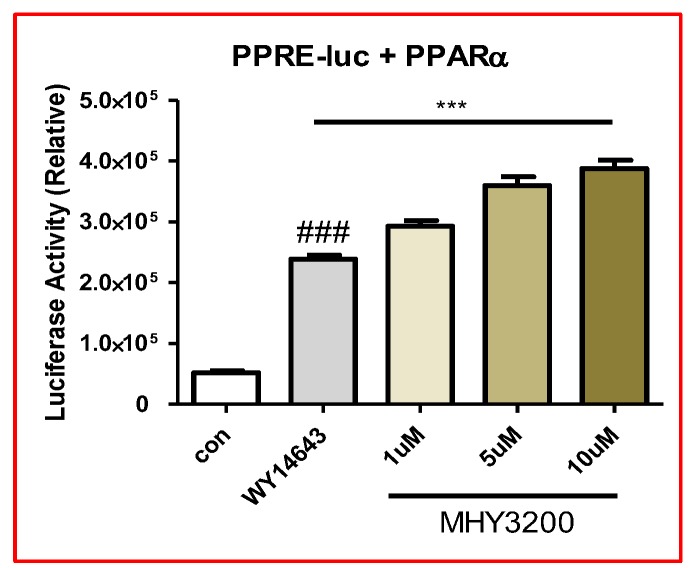
Effects of MHY3200 on peroxisome proliferator-activated receptor α (PPARα) activity. Transcriptional activity of PPARα by using a reporter gene assay in AC2F cells. The data represent the mean ± SEM. One-way analysis of variance (ANOVA) was used to determine the statistical significance of the results: ^###^ *p* < 0.001 versus control, *** *p* < 0.001 versus WY14643.

As shown Figure 1, the synthesis of 2-(4-(5-chlorobenzo[*d*]thiazol-2-yl)phenoxy)-2,2-difluoroacetic acid (MHY3200) was described. To evaluate whether MHY3200 induced the transcriptional activity of PPARα, a luciferase binding assay was performed in AC2F cells. The transcriptional activity of PPARα was significantly increased by MHY3200 in a dose-dependent manner (Figure 2). Also, the predicted binding affinity of MHY3200 and PPARα was −8.89 kcal/mol in the AutoDock 4.2 program and −34.2 kcal/mol in the Dock6 program, respectively, and the binding affinity of WY14643 for PPARα was −8.44 kcal/mol, respectively (data not shown). These data showed that MHY3200 was a potential PPARα agonist.

### 2.2. Biochemical Analyses

To investigate the alterations, metabolic parameters of HFD rats were measured. Table 1 lists the changes in body weight, food intake, and water intake during the experimental period. The HFD rats showed significant weight gain, but this was inhibited slightly, though not significantly, by the administration of MHY3200. The levels of food and water intake were not affected by MHY3200. Moreover, the serum TG level increased in the HFD rats compared to the chow rats. This increase was inhibited by the administration of MHY3200. Also, the serum glucose, ALT, and insulin levels all increased in the HFD rats compared to the chow rats. However, this increase was significantly inhibited by the administration of MHY3200. Moreover, the serum FFA and AST levels increased in the HFD rats, but, this increase was inhibited slightly by the administration of MHY3200.

### 2.3. Effects of MHY3200 on Hepatic Lipid Accumulation in the HFD Rat Liver

**Figure 3:**
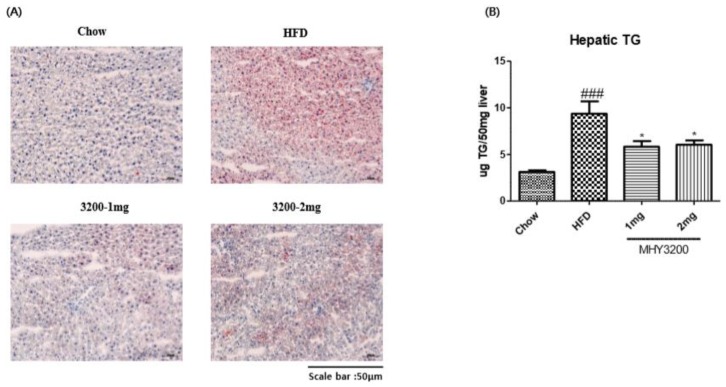
MHY3200 improved hepatic lipid accumulation in the liver of high-fat diet (HFD) rats. (**A**) Oil-red O staining. (**B**) Hepatic triglyceride contents. The data represent the mean ± SEM. One-way analysis of variance (ANOVA) was used to determine the statistical significance of the results: ^###^ *p* < 0.001 vs. chow, * *p* < 0.05 vs. HFD.

**Figure 4:**
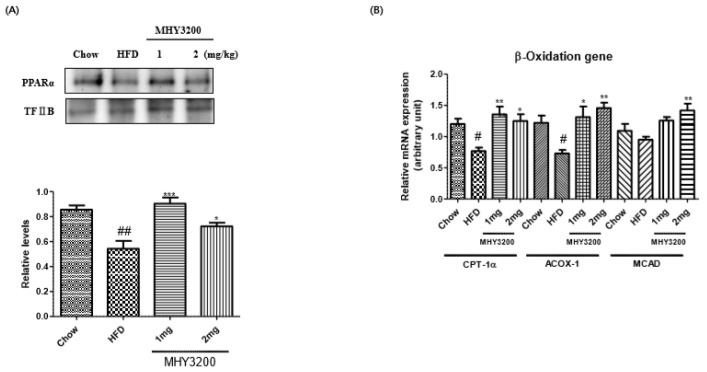
Effects of MHY3200 on PPARα protein expression, beta-oxidation genes, and mRNA levels in the liver of HFD rats. (**A**) Protein levels of PPARα in the nucleus. TFIIB was used as an internal nuclear control. One representative blot of each protein is shown from three independent experiments in each group that yielded similar results. (**B**) mRNA expression of beta-oxidation genes were evaluated by using RT-PCR. The results were normalized to the expression of a reference gene (GAPDH). The data represent the mean ± SEM. One-way analysis of variance (ANOVA) was used to determine the statistical significance of the results: ^#^ *p* < 0.05, ^##^ *p* < 0.01 vs. Chow, * *p* < 0.05, ** *p* < 0.01, *** *p* < 0.001 vs. HFD. TFIIB, Transcription Factor II B

Our aim was to determine whether MHY3200 improved HFD-induced fatty liver. As shown in Figure 3A, Oil Red O staining indicated lipid droplet accumulation in the liver of HFD rats, which was markedly decreased by MHY3200 administration. To verify the preventative effects of MHY3200 on lipid accumulation, we examined hepatic TG content. As shown in Figure 3B, hepatic TG content was higher in the liver of HFD rats than in chow-fed rats, However, MHY3200 significantly decreased the hepatic TG levels in HFD rats. PPARs play major roles in various biological processes, such as cell proliferation, insulin sensitivity, and lipid metabolism. Among the three subtypes of PPARs, PPARα improved lipid accumulation through the activation of β-oxidation gene expression [18,19]. We examined the effect of MHY3200 on PPARα expression using western blot analysis. As shown in Figure 4A, PPARα activation was reduced in HFD rats—however, MHY3200 administration increased PPARα protein levels, and expression of lipid oxidation-related genes (CPT-1α, ACOX-1, and MCAD) increased (Figure 4B). These results suggested that MHY3200 improves hepatic TG accumulation through an increase in PPARα activation and β-oxidation genes. 

### 2.4. Effects of MHY3200 on ER Stress, Insulin Signaling and Inflammation in the HFD Rat Liver

**Figure 5:**
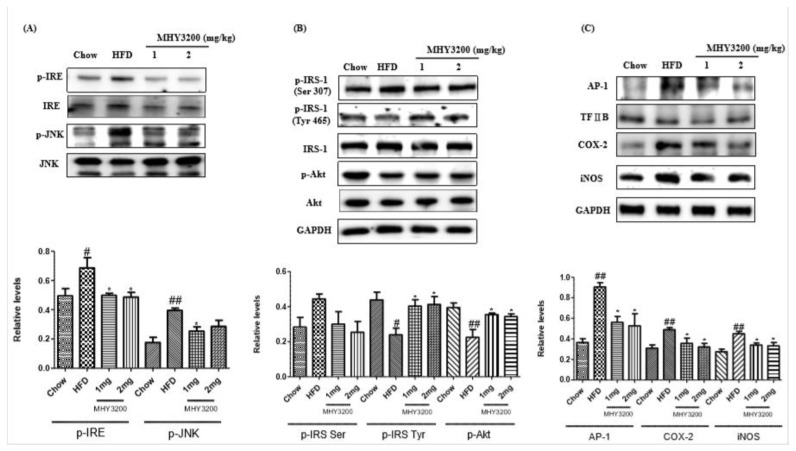
Effects of MHY3200 on endoplasmic reticulum (ER) stress, insulin signaling, and inflammation in the livers of HFD rats. (**A**) The ER stress markers, IRE, JNK, (**B**) phosphorylated IRS-1 (Ser 307, Tyr 465) and phosphorylated Akt, and (**C**) inflammatory genes (nucleus AP-1, COX-2, and iNOS) were analyzed by using western blotting. One representative blot of each protein is shown from three independent experiments in each group that yielded similar results. TFIIB and GAPDH were amplified as the nuclear and cytosolic internal controls, respectively. The data represent the mean ± SEM. One-way analysis of variance (ANOVA) was used to determine the statistical significance of the results: ^#^ *p* < 0.05, ^##^ *p* < 0.01, vs. chow, * *p* < 0.05, vs. HFD. IRE, inositol-requiring kinase; JNK, c-Jun N-terminal kinase; IRS-1, insulin receptor substrate 1; Akt, Protein kinase B.

As shown in Figure 5A, the protein levels of IRE and JNK phosphorylation were higher in the HFD group than in the chow diet group, whereas MHY3200 administration reduced them. Activated JNK led to phosphorylation of a serine residue (Ser 307) of IRS and decreased the phosphorylation of tyrosine residues (Tyr 465) of IRS, as well as of Akt, in the HFD group, whereas the MHY3200-fed groups showed attenuated expression levels of the insulin-signaling pathway-related molecules (Figure 5B). Moreover, the protein expression of AP-1 was higher in the HFD groups than in the chow-fed groups, and the MHY3200-treated rats showed a decreased level of AP-1. The expressions of inflammation-related proteins, such as COX-2 and iNOS, increased in the HFD groups, but was reduced by MHY3200 administration (Figure 5C). These data suggested that MHY3200 improved insulin signaling and inflammation through the inhibition of IRE/JNK expression.

## 3. Discussion

The worldwide prevalence of obesity is a major public health problem—thus, a therapeutic approach that prevents fatty liver disease by using anti-obesity agents which improve insulin resistance and hyperlipidemia is needed [20]. Obesity is closely related to an impaired glucose and lipid metabolism, which can cause various metabolic diseases, such as fatty liver, dyslipidemia, and type 2 diabetes. Prolonged exposure to a high-fat diet and diet-induced obesity causes inflammatory responses and lipid accumulation, which may lead to glucose intolerance and insulin resistance [21]. In a previous study, an animal model of HFD-induced NAFLD has been widely used to identify the pathogenesis of and evaluate novel treatments for NAFLD [22,23]. In addition, it was found that HFD administration induced ER stress, insulin resistance, and inflammation in obese rats. 

In the present assay, MHY3200 administration significantly decreased TG and insulin levels, and slightly reduced free fatty acid and glucose levels. The administration of MHY3200 had lower plasma AST and ALT levels compared with HFD groups, with the MHY3200 administration group having the lowest levels among the groups. Therefore, these results suggest that MHY3200 has a protective effect against hepatic injury. A previous report showed that MHY908 is a potent dual agonist of PPARα/γ that improves ER stress and insulin sensitivity by PPARα and PPARγ activation in the liver and adipose tissue, respectively [24]. Consistent with this study, MHY3200 was found to be a PPARα agonist based on a PPRE-luciferase assay in AC2F cells, and acts as a PPARα agonist in the liver. MHY3200 administration reduced HFD-induced hepatic TG accumulation through PPARα activation, and β-oxidation genes were increased by MHY3200 administration. 

Many studies have documented that PPARα negatively regulates inflammatory responses [25]. Thus, we examined the anti-inflammatory effect of MHY3200 on the liver of HFD rats. Although the molecular mechanisms are unclear, it is likely that MHY3200 improved inflammatory mediators in the liver of HFD rats. This study supported previous data that MHY3200 improved inflammatory responses through the activation of PPARα in the liver. Various studies have reported that ER stress is related to the progression of insulin resistance and inflammation in obesity by an excess of lipids, causing the activation of the ER stress response. In addition, fatty liver is closely involved with insulin resistance, fibrosis, and apoptosis, as well as an increased inflammatory response [26]. Many studies have reported that the activation of PPARα is related to a reduction in inflammatory gene expression and the inhibition of AP-1 signaling [27,28]. In a previously identified study, it was found that the effect of 4-(benzo[*d*]thiazol-2-yl)benzene-1,3-diol (MHY553), a novel PPARα agonist, alleviated hepatic steatosis through an increase of fatty acid oxidation and a decrease in inflammation in aged rats [13]. This study identified that MHY3200 suppressed IRE-1, JNK, and AP-1 activation, as well as the HFD-induced inflammatory response, including COX-2 and iNOS. Collectively, these results suggest that MHY3200 suppresses hepatic inflammation through a direct or indirect effect of PPARα activation.

## 4. Materials and Methods

### 4.1. Reagents

The chemical was dissolved in dimethyl sulfoxide (DMSO). Chemical reagents were obtained from Sigma (St. Louis, MO, USA). All primary and secondary antibodies used for immunoblotting were used at dilutions of 1:1000 and 1:10,000, respectively. The antibody against p-IRE (Ser 724) was obtained from Thermo Fisher Scientific (Waltham, MA, USA), the antibody against PPARα was purchased from Abcam (Cambridge, UK), and other antibodies were obtained from Santa Cruz Biotechnology (Santa Cruz, CA, USA). Anti-rabbit IgG-horseradish peroxidase-conjugated antibodies, anti-goat IgG-horseradish peroxidase-conjugated antibodies, and anti-mouse IgG-horseradish peroxidase-conjugated antibodies were obtained from Santa Cruz Biotechnology.

### 4.2. General

All chemical reagents were commercially available and used without further purification. Low-resolution mass spectrometry (MS) and high-resolution MS data were obtained on an Expression CMS (Advion, Ithaca, NY, USA) and a 6530 Accurate Mass quadrupole time-of-flight liquid-chromatography mass spectrometer (Agilent), respectively. Nuclear magnetic resonance (NMR) data were recorded on a Varian Unity INOVA 400 spectrometer and Varian Unity AS500 spectrometer (Agilent Technologies, Santa Clara, CA, USA) using CDCl_3_, and DMSO-*d*_6_ and chemical shifts were reported in parts per million (ppm) with reference to the respective residual solvent or deuterated peaks (δ_H_ 7.24 and δ_C_ 77.0 for CDCl_3_, δ_H_ 2.50 and δ_C_ 39.7 for DMSO-*d*_6_). Coupling constants are reported in hertz. The abbreviations used are as follows: s (singlet), d (doublet), t (triplets), q (quartet), or dd (doublet of doublets). All the reactions described below were performed under a nitrogen atmosphere and monitored by thin-layer chromatography (TLC). TLC was performed on Merck precoated 60F_254_ plates. All anhydrous solvents were distilled over CaH_2_ or Na/benzophenone prior to use. The identities of all final compounds was confirmed by 1D (^1^H and ^13^C) and low- and high-resolution mass spectrometry.

#### 4.2.1. Synthesis of Ethyl 2,2-difluoro-2-(4-formylphenoxy)acetate (Compound **1**)

To a stirred solution of 4-hydroxybenzaldehyde (2.0 g, 16.39 mmol) and DBU (1,8-diazabicyclo[5.4.0]undec-7-ene, 2.94 mL, 19.65 mmol) in anhydrous THF (tetrahydrofuran, 30 mL), was added ethyl bromodifluoroacetate (2.52 mL, 19.65 mmol) dropwise at room temperature. Then the reaction mixture was stirred at 60 °C for 2 h, and partitioned between diethyl ether and water. The organic layer was dried over anhydrous MgSO_4_, filtered, and evaporated under reduced pressure. The resultant residue was purified by silica gel column chromatography using hexanes and ethyl acetate (5:1) as an eluent to give compound **1** (2.45 g, 61.3%) as a colorless liquid. ^1^H-NMR (400 MHz, CDCl_3_) *δ* 9.99 (s, 1 H, CHO), 7.91 (d, 2 H, *J* = 8.4 Hz, 3-H, 5-H), 7.37 (d, 2 H, *J* = 8.4 Hz, 2-H, 6-H), 4.40 (q, 2 H, *J* = 7.2 Hz, C*H_2_*CH_3_), 1.37 (t, 3 H, *J* = 7.2 Hz, CH_2_C*H_3_*); ^13^C-NMR (100 MHz, CDCl_3_) *δ* 191.0, 159.7 (t, *J* = 40.5 Hz), 154.4, 134.3, 131.7, 121.7, 114.4 (t, *J* = 273.4 Hz), 64.3. 14.1; HRMS (ESI+) *m/z* C_11_H_11_F_2_O_4_ (M + H)^+^ Calcd 245.0620, obsd 245.0622.

#### 4.2.2. Synthesis of Ethyl 2-(4-(5-chlorobenzo[d]thiazol-2-yl)phenoxy)-2,2-difluoroacetate (Compound **2**)

To a stirred solution of compound **1** (500 mg, 2.05 mmol) and 2-amino-4-chlorobenzenethiol (326.9 mg, 2.05 mmol) in anhydrous DMF (*N*,*N*-dimethylformamide, 5.0 mL) was added Na_2_S_2_O_5_ (468 mg, 2.46 mmol), and the reaction mixture was stirred at 80 °C for 12 h. After cooling, the reaction mixture was partitioned between diethyl ether and water. The organic layer was dried over anhydrous MgSO_4_, filtered, and evaporated under reduced pressure. The resulting residue was purified by silica gel column chromatography using hexanes and ethyl acetate (5:1) as an eluent to give compound **2** (689 mg, 87.7%). ^1^H-NMR (400 MHz, CDCl_3_) *δ* 8.08 (d, 2 H, *J* = 8.8 Hz, 3-H, 5-H), 8.04 (d, 1 H, *J* = 2.4 Hz, 4′-H), 7.80 (d, 1 H, *J* = 8.8 Hz, 7′-H), 7.37 (dd, 1 H, *J* = 8.8, 2.0 Hz, 6′-H), 7.35 (d, 2 H, *J* = 8.4 Hz, 2-H, 6-H), 4.41 (q, 2 H, *J* = 6.8 Hz, C*H_2_*CH_3_), 1.38 (t, 3 H, *J* = 7.2 Hz, CH_2_C*H_3_*); ^13^C-NMR (100 MHz, CDCl_3_) *δ* 168.6, 160.0 (t, *J* = 41.6 Hz), 155.1, 152.0, 133.6, 132.7, 131.5, 129.2, 126.1, 123.3, 122.6, 122.2, 114.2 (t, *J* = 273.0 Hz), 64.2, 14.1; HRMS (ESI+) *m/z* C_17_H_13_ClF_2_NO_3_S (M + H)^+^ Calcd 384.0267, obsd 384.0273.

#### 4.2.3. Synthesis of 2-(4-(5-chlorobenzo[d]thiazol-2-yl)phenoxy)-2,2-difluoroacetic Acid (MHY3200)

To a stirred solution of compound **2** (532 mg, 1.39 mmol) in 1,4-dioxane (4.6 mL) was added 1N NaOH aqueous solution (2.78 mL, 2.78 mmol). The reaction mixture was stirred at room temperature for 25 h, and partitioned between methylene chloride and water. The aqueous layer was acidified to pH 2 with 1M HCl, and the precipitates generated were filtered to give MHY3200 (480.4 mg, 97.4%) as a white solid. ^1^H-NMR (400 MHz, DMSO-*d_6_*) *δ* 8.18 (d, 1 H, *J* = 8.4 Hz, 7′-H), 8.14 (d, 2 H, *J* = 8.4 Hz, 3-H, 5-H), 8.13 (s, 1 H, 4′-H), 7.50 (d, 1 H, *J* = 8.4 Hz, 6′-H), 7.41 (d, 2 H, *J* = 8.4 Hz, 2-H, 6-H); ^13^C-NMR (125 MHz, DMSO-*d_6_*) *δ* 159.4, 151.3 (t, *J* = 38.5 Hz), 145.4, 142.7, 124.4, 122.5, 121.4, 120.2, 116.8, 115.0, 113.3, 112.7, 106.4 (t, *J* = 272.3 Hz); LRMS (ESI+) *m/z* 356 (M + H)^+^; HRMS (ESI+) *m/z* C_15_H_9_ClF_2_NO_3_S (M+H)^+^ Calcd 355.9954, obsd 355.9959.

### 4.3. Cell Culture System

The AC2F rat hepatocyte cell line was obtained from the ATCC (American Type Culture Collection, Rockville, MD, USA). Cells were cultured in Dulbecco’s Modified Eagle Medium (DMEM, Welgene, Gyeongsan, Gyeongsangbuk-do, Korea) supplemented with 10% heat-inactivated (56 °C for 30 min) fetal bovine serum (FBS, Welgene), 100 U/mL penicillin, and 100 μg/mL streptomycin (Welgene). The cells were maintained at 37 °C in a humidified atmosphere with a 5% CO_2_ humidified atmosphere. The medium was replaced with fresh medium after 1 day to remove non-adherent cells or cell debris.

### 4.4. Luciferase Assay

The luciferase assay was conducted in 96-well plates. The AC2F cells were seeded and subsequently transfected with Lipofectamine 3000 transfection reagent (Invitrogen, Grand Island, NY, USA) and a plasmid-containing PPRE-the-luciferase reporter vector (0.1 μg), which was a kind gift from Dr. Christoper K. Glass of the University of California, San Diego, CA, and a PPAR expression vector (0.01 μg), which was a kind gift from Dr. Han Geuk Seo of Konkuk University, Seoul, Korea. After 24 h, the cells were treated with MHY3200 and WY14643 (10 μM), respectively, for 5 h as positive controls for PPARα activation. Luciferase activity was detected by using the One-Glo luciferase assay system (Promega, Madison, WI, USA) and measured by using a luminometer (Berthold Technologies GmbH & Co., Bad Wildbad, Germany).

### 4.5. Animal Experiments

To estimate the effect of MHY3200 on hepatic lipid accumulation, insulin resistance, and inflammation in HFD rat livers, 6-month-old Sprague Dawley male rats were purchased from Samtako (Gueonggi-do, Korea) and acclimated to the animal care facility for 7 days before the experiments. The animals were housed in an air-conditioned atmosphere with a 12 h light/dark cycle and given free access to standard rodent chow (Samtako) and water. The animal study was approved by the Institutional Animal Care Committee of Pusan National University and performed in accordance with the guidelines for animal experiments issued by Pusan National University (Approval Number PNU-2015-0759). The rats (*n* = 6 per groups) were treated with vehicle or MHY3200 (1 or 2 mg/kg/day) by oral gavage and administered either a normal diet or a 60% fat diet (HFD) for 4 weeks. Food intake was monitored daily, and body weight was measured once every 5 days.

### 4.6. Serum Biochemical Analyses

After the animals were sacrificed, blood samples were collected. Serum samples were prepared by centrifugation (4 °C, 2000× *g* for 15 min). Serum glucose and TG levels were analyzed by using kits from Bioassay Systems (Hayward, CA, USA), and free fatty acid, ALT, and AST levels were analyzed by using kits from Asan Pharmaceuticals Co., Ltd. (Asan, South Korea). To measure the serum insulin levels, the rat ELISA kit (SHIBAYAGI, Shibukawa, Japan) was used.

### 4.7. Hepatic TG Measurement

Liver tissues were homogenized in phosphate-buffered saline (PBS). TGs were extracted in a methanol and chloroform (1:2) solution at room temperature for 2 h. After the elimination of impurities by using a filter, the TG liquid solvent was dried. TG levels were then evaluated by using a kit (Bioassay Systems, Hayward, CA, USA).

### 4.8. Tissue Protein Extraction

All solutions, tubes, and centrifuges were maintained at 0–4 °C. Liver tissue (1 g) was homogenized with 700 μL hypotonic lysis buffer [buffer A: 100 mM Tris (pH 7.4), 20 mM β-glycerophosphate, 20 mM NaF, 2 mM sodium orthovanadate, 1 mM EDTA, 0.01 mM DTT, 0.5 mM PMSF, 1 μM pepstatin, 2 μM leupeptin, and 10 mM HEPES (pH 7.8)] by using a tissue homogenizer for 30 s. The homogenates were kept on ice for 20 min, after which 125 μL of 10% Nonidet P-40 (NP-40) solution was added, mixed for 15 s, and centrifuged at 12,000× *g* for 15 min at 4 °C. The supernatant was collected and used as the cytosolic fraction. The pellets were washed with 300 μL hypotonic buffer A and 25 μL of 10% NP-40, centrifuged, suspended in 200 μL buffer C [50 mM KCl, 300 mM NaCl, 1 mM DTT, 0.1 mM EDTA, 0.1 mM PMSF, 10% (*v*/*v*) glycerol, 1 μM pepstatin, 2 μM leupeptin, 20 mM β-glycerophosphate, 20 mM NaF, 2 mM Na-orthovanadate, and 50 mM HEPES (pH 7.8)], kept on ice for 30 min, and centrifuged at 14,000× *g* for 10 min. The supernatant, which contained nuclear proteins, was collected, divided into aliquots, and stored at −80 °C until used. The protein concentration was measured by using the bicinchoninic acid (BCA) assay method with bovine serum albumin (BSA) as a standard.

### 4.9. Western Blot Analysis

As described previously, western blotting was performed [29]. The lysed samples were mixed in a 1:1 ratio with gel-loading buffer [0.125 M Tris-HCl, pH 6.8, 4% sodium dodecyl sulfate (SDS), 10% 2-mercaptoethanol, and 0.2% bromophenol blue] and boiled for 5 min. Equal amounts of protein were separated by SDS-polyacrylamide gel electrophoresis on a 6–17% acrylamide gel. The gels were subsequently transferred onto Immobilon-P transfer membranes (Millipore Corp, Bedford, MA, USA). The membranes were immediately incubated in blocking buffer [5% non-fat dry milk in TBS-Tween (TBS-T) buffer containing 10 mM Tris, 100 mM NaCl, and 0.1% Tween 20, pH 7.5] at room temperature for 1 h. Next, the membranes were washed in TBS-T buffer for 30 min and incubated at 4 °C overnight with the appropriate specific primary antibodies. After 30 min washing in TBS-T buffer, the membranes were incubated with an appropriate secondary antibody at room temperature for 1 h. Subsequently, after washing in TBS-T buffer for 40 min, antibody labeling was detected by the application of Enhanced Chemiluminescence (ECL) in accordance with the manufacturer’s instruction and exposure to a radiographic film.

### 4.10. Isolation and Quantitative Real-Time PCR

Tissue RNA was purified by using the RiboEx Total RNA (Geneall, Korea) in accordance with the manufacturer’s instructions. Total RNA (2.0 μg) resolved in RNase-free water was reverse-transcribed by using a cDNA synthesis kit (Geneall). Quantitative real-time PCR was performed by using the SensiFAST SYBT NO-ROS kit (BIOLINE, London) and a CFX Connect System (Bio-Rad Laboratories, Inc., Hercules, CA, USA).

### 4.11. Statistical Analysis

All results were expressed as the mean ± standard error of the mean (SEM). Treatments were compared by using a one-way analysis of variance (ANOVA) test, followed by the Bonferroni test. Statistical significance was accepted for *p* values of <0.05. The analyses were computed by using GraphPad Prism 5 (GraphPad Software, La Jolla, CA, USA).

## 5. Conclusions

**Figure 6:**
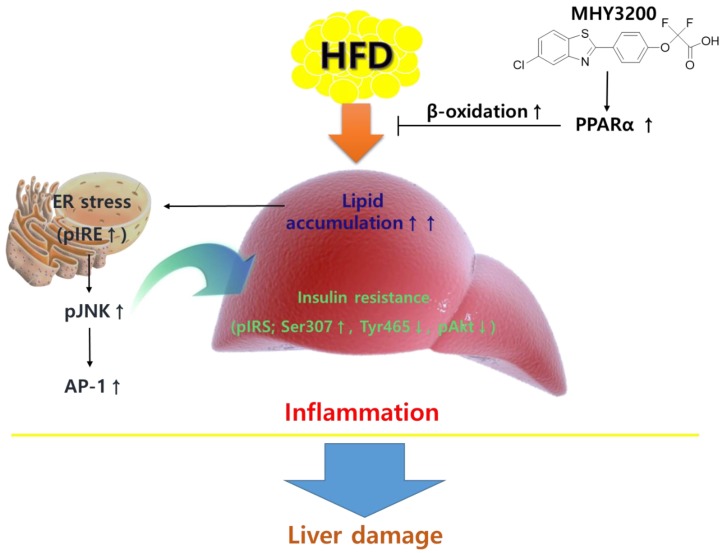
A possible mechanism of action of MHY3200 in HFD rat liver tissues. MHY3200 exerted beneficial effects on lipid accumulation-independent, ER stress- and insulin resistance-induced inflammation in the livers of rats with high-fat diet-induced obesity.

This study focused on the effects of lipid accumulation-, ER stress-, and insulin resistance-related inflammation in the livers of the HFD rats treated with MHY3200. The present study showed that the administration of MHY3200 to HFD rats had beneficial effects on the prevention of NAFLD—at least in part—by ameliorating the lipid accumulation-induced inflammation. It was shown that MHY3200 inhibited the lipid accumulation-related ER stress (IRE) expression in the liver. Although the detailed mechanisms of MHY3200 was not clarified in the present study, the findings suggest therapeutic evidence for the favorable influence of MHY3200 on reversing the effects of HFD-related liver damage. This study demonstrated that MHY3200 improved hepatic lipid accumulation, insulin resistance, and inflammation through increased PPARα activation in HFD rats (Figure 6). Therefore, it can be said that MHY3200 is a potential pharmaceutical agent that may ameliorate metabolic syndrome derived from diet-induced obesity.

## Figures and Tables

**Table 1:** Biochemical analyses.

				HFD		
Item		Chow		Con	1 mg	2 mg
Bodyweight						
Initial (g)		289.3 ± 2.7		289.5 ± 3.5	290.2 ± 4.2	286.3 ± 2.8
Final (g)		448 ± 9.2 ^a^		538.3 ± 18.1	506.2 ± 9.2	515 ± 15.5
Gain (g)		158.7 ± 8.8 ^c^		250.1 ± 21.1	216 ± 8	228.7 ± 14.5
Food Intake (g/days)		22.2 ± 1.4		18.5 ± 1.4	20.3 ± 1.7	19.6 ± 1.6
Water Intake (mL/day)		51.7 ± 12.9		69.1 ± 16.1	44.8 ± 12.3	79.3 ± 14.9
Serum ALT (IU/L)		1.9 ± 0.5 ^b^		3.7 ± 0.3	1.7 ± 0.2 ^c^	1.7 ± 0.2 ^c^
Serum AST (IU/L)		36.6 ± 3.2		39.1 ± 1.1	26.8 ± 3.4	25.5 ± 2.3
Serum Triglyceride (mg/dL)		61.1± 1.6 ^a^		81.2 ± 7.7	59.8 ± 5.1 ^a^	53.3 ± 2.1 ^b^
Serum Cholesterol (mg/dL)		96.9 ± 4.9 ^c^		207.8 ± 11.2	162 ± 16.9 ^b^	317.9 ± 32.3 ^a^
Serum FFA (uEq/L)		427 ± 32.1 ^b^		644 ± 20	627 ± 55.1	548.6 ± 28.9
Serum Glucose (mg/dL)		91.8 ± 3.1 ^a^		103.3 ± 2.29	95.3 ± 2.17	91 ± 2.9 ^a^
Serum Insulin (ng/mL)		1.3 ± 0.08 ^a^		2.1 ± 0.24	1.3 ± 0.13 ^a^	0.99 ± 0.05 ^b^

*Note;* Data are expressed as mean ± SEM. Significance: ^a^ *p* < 0.05, ^b^ *p* < 0.01, ^c^ *p* < 0.001 vs. Con.

## References

[1] Marchesini G., Bugianesi E., Forlani G., Cerrelli F., Lenzi M., Manini R., Natale S., Vanni E., Villanova N., Melchionda N. (2003). Nonalcoholic fatty liver, steatohepatitis, and the metabolic syndrome. Hepatology.

[2] Schneider A.L., Lazo M., Selvin E., Clark J.M. (2014). Racial differences in nonalcoholic fatty liver disease in the U.S. population. Obesity.

[3] Poonawala A., Nair S.P., Thuluvath P.J. (2000). Prevalence of obesity and diabetes in patients with cryptogenic cirrhosis: A case-control study. Hepatology.

[4] Cai D., Yuan M., Frantz D.F., Melendez P.A., Hansen L., Lee J., Shoelson S.E. (2005). Local and systemic insulin resistance resulting from hepatic activation of IKK-beta and NF-kappaB. Nat. Med..

[5] Mendez-Sanchez N., Arrese M., Zamora-Valdes D., Uribe M. (2007). Current concepts in the pathogenesis of nonalcoholic fatty liver disease. Liver Int..

[6] Roberts C.K., Hevener A.L., Barnard R.J. (2013). Metabolic syndrome and insulin resistance: Underlying causes and modification by exercise training. Compr. Physiol..

[7] Salvado L., Palomer X., Barroso E., Vazquez-Carrera M. (2015). Targeting endoplasmic reticulum stress in insulin resistance. Trends Endocrinol. Metab..

[8] Boden G. (2011). Obesity, insulin resistance and free fatty acids. Curr. Opin. Endocrinol. Diabetes Obes..

[9] Haynes C.M., Titus E.A., Cooper A.A. (2004). Degradation of misfolded proteins prevents ER-derived oxidative stress and cell death. Mol. Cell.

[10] Wellen K.E., Hotamisligil G.S. (2005). Inflammation, stress, and diabetes. J. Clin. Investig..

[11] Ozcan U., Cao Q., Yilmaz E., Lee A.H., Iwakoshi N.N., Ozdelen E., Tuncman G., Görgün C., Glimcher L.H., Hotamisligil G.S. (2004). Endoplasmic reticulum stress links obesity, insulin action, and type 2 diabetes. Science.

[12] Ip Y.T., Davis R.J. (1998). Signal transduction by the c-Jun N-terminal kinase (JNK)—From inflammation to development. Curr. Opin. Cell Biol..

[13] Issemann I., Green S. (1990). Activation of a member of the steroid hormone receptor superfamily by peroxisome proliferators. Nature.

[14] Jay M.A., Ren J. (2007). Peroxisome proliferator-activated receptor (PPAR) in metabolic syndrome and type 2 diabetes mellitus. Curr. Diabetes Rev..

[15] Chan S.M., Sun R.Q., Zeng X.Y., Choong Z.H., Wang H., Watt M.J., Ye J.M. (2013). Activation of PPARα ameliorates hepatic insulin resistance and steatosis in high fructose–fed mice despite increased endoplasmic reticulum stress. Diabetes.

[16] Park M.H., Park J.Y., Lee H.J., Kim D.H., Park D., Jeong H.O., Park C.H., Chun P., Moon H.R., Chung H.Y. (2013). Potent anti-diabetic effects of MHY908, a newly synthesized PPAR alpha/gamma dual agonist in db/db mice. PLoS ONE.

[17] Kim S.M., Lee B., An H.J., Kim D.H., Park K.C., Noh S.G., Chung K.W., Lee E.K., Kim K.M., Kim D.H. (2017). Novel PPARalpha agonist MHY553 alleviates hepatic steatosis by increasing fatty acid oxidation and decreasing inflammation during aging. Oncotarget.

[18] Guan Y., Zhang Y., Breyer M.D. (2002). The role of PPARs in the transcriptional control of cellular processes. Drug News Perspect..

[19] Ferre P. (2004). The biology of peroxisome proliferator-activated receptors: Relationship with lipid metabolism and insulin sensitivity. Diabetes.

[20] Kopelman P.G. (2000). Obesity as a medical problem. Nature.

[21] Hotamisligil G.S., Shargill N.S., Spiegelman B.M. (1993). Adipose expression of tumor necrosis factor-alpha: Direct role in obesity-linked insulin resistance. Science.

[22] Barbuio R., Milanski M., Bertolo M.B., Saad M.J., Velloso L.A. (2007). Infliximab reverses steatosis and improves insulin signal transduction in liver of rats fed a high-fat diet. J. Endocrinol..

[23] Samuel V.T., Liu Z.X., Qu X., Elder B.D., Bilz S., Befroy D., Romanelli A.J., Shulman G.I. (2004). Mechanism of hepatic insulin resistance in non-alcoholic fatty liver disease. J. Biol. Chem..

[24] Park M.H., Kim D.H., Kim M.J., Lee E.K., An H.J., Jeong J.W., Kim H.R., Kim S.J., Yu B.P., Moon H.R. (2016). Effects of MHY908, a new synthetic PPARalpha/gamma dual agonist, on inflammatory responses and insulin resistance in aged rats. J. Gerontol. A Biol. Sci. Med. Sci..

[25] Pawlak M., Lefebvre P., Staels B. (2015). Molecular mechanism of PPARalpha action and its impact on lipid metabolism, inflammation and fibrosis in non-alcoholic fatty liver disease. J. Hepatol..

[26] Bertolotti M., Lonardo A., Mussi C., Baldelli E., Pellegrini E., Ballestri S., Romagnoli D., Loria P. (2014). Nonalcoholic fatty liver disease and aging: Epidemiology to management. World J. Gastroenterol..

[27] Poulsen L., Siersbaek M., Mandrup S. (2012). PPARs: Fatty acid sensors controlling metabolism. Semin. Cell Dev. Biol..

[28] Vanden Berghe W., Vermeulen L., Delerive P., De Bosscher K., Staels B., Haegeman G. (2003). A paradigm for gene regulation: Inflammation, NF-kappaB and PPAR. Adv. Exp. Med. Biol..

[29] Gershoni J.M., Palade G.E. (1983). Protein blotting: Principles and applications. Anal. Biochem..

